# Poisoning by Tea

**Published:** 1889-08

**Authors:** E. W. Berridge


### POISONING BY TEA.

E. W. BERRIDGE, M. D.

In 1880 a colleague in the United States communicated to me the following curious effect of tea: A woman, aged about thirty-three, had been married twice. Always during her married life had the natural sexual desire and pleasure, till about eight years ago, when she began to eat large quantities of tea-leaves, sometimes dry, but generally boiled. She chiefly used the Japan tea. Since then face and skin have become sallow and greenish. She has still the same sexual desire, but the orgasm occurs at the first touch of coitus, after which there is no pleasure; and if the act is long continued, she feels a bearing-down. The touch of another person's hand to her head will have the same effect. She never had these symptoms before she took the tea.

*Berberis* has the reverse symptom (see Hering's *Guiding Symptoms*).

